# A Tet/Q Hybrid System for Robust and Versatile Control of Transgene Expression in *C. elegans*

**DOI:** 10.1016/j.isci.2018.12.023

**Published:** 2018-12-27

**Authors:** Shaoshuai Mao, Yingchuan Qi, Huanhu Zhu, Xinxin Huang, Yan Zou, Tian Chi

**Affiliations:** 1 School of Life Sciences and Technology, ShanghaiTech University, Shanghai, P.R. China; 2 Department Immunobiology, Yale University School of Medicine, New Haven, CT, USA

**Keywords:** Genetics, Techniques in Genetics, Model Organism

## Abstract

Binary gene regulatory tools such as the Tetracycline (Tet)-controlled transcription system have revolutionized genetic research in multiple organisms, but their applications to the worm remain very limited. Here we report that the canonical Tet system is largely inactive in the worm but can be adapted for the worm by introducing multiple modifications, a crucial one being the use of the transcription activation domain from the fungal Q binary system. The resultant Tet/Q hybrid system proves more robust and flexible than either of its precursors, enabling elaborate modes of transgene manipulation previously hard to achieve in the worm, including *inducible* intersectional regulation and, in combination with the Q system, independent control of distinct transgenes within the same cells. Furthermore, we demonstrated, as an example of its applications, that the hybrid system can tightly and efficiently control Cre expression. This study establishes Tet/Q as a premier binary system for worm genetic research.

## Highlights

• The popular Tet-controlled gene regulatory system proves inapplicable to the worm
• The fungal Q binary gene regulatory system is moderately active in the worm
• A hybrid Tet/Q system is capable of robust, rapid and tunable transgene induction
• Further modifications enable sophisticated regulation previously hard to achieve

## Graphical Abstract

**Figure:**
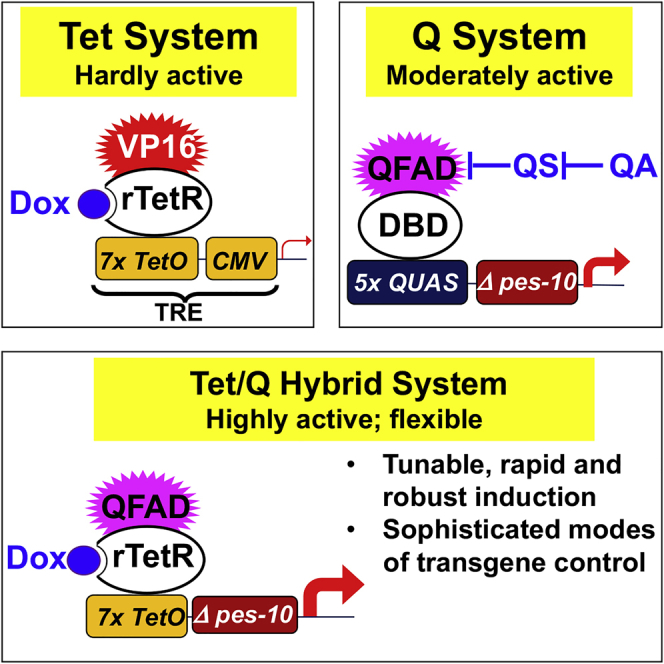

## Introduction

**Figure 1:**
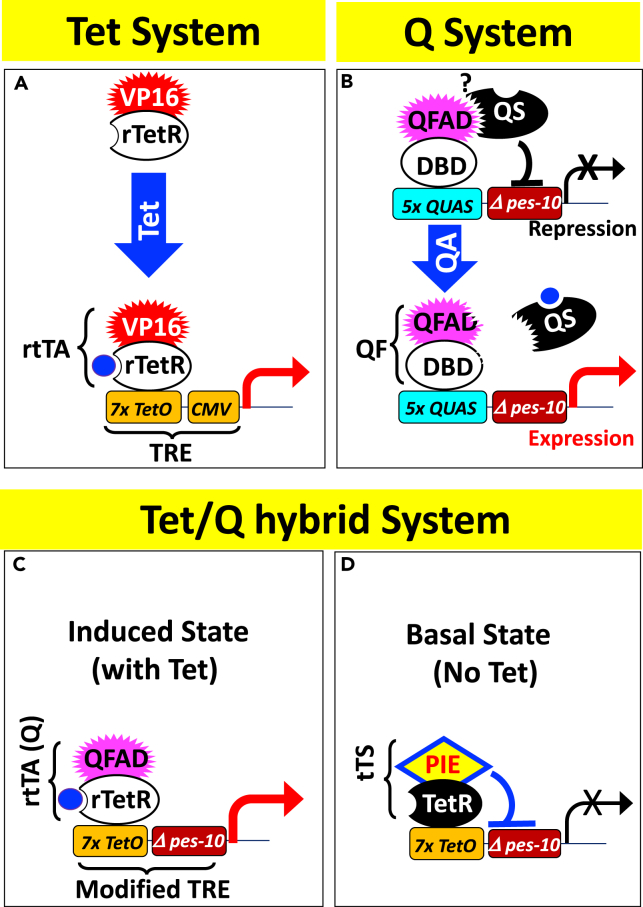
Three Binary Gene Regulatory Systems, Each Containing an Activator and a Responder (A) The canonical Tet system (Schonig et al., 2010). The activator consists of the VP16 activation domain fused to bacterial Tet repressor (TetR, not shown) or its derivative reverse TetR (rTetR), which recognizes the tet operator (*tetO*) in the absence and presence of tetracycline (Tet), respectively. The Tetracycline Response Element (TRE) comprises seven tandem repeats of *tetO* upstream of a *cmv* minimal promoter. rtTA, **r**everse **t**et-regulated **T**ranscription **A**ctivator. (B) The Q system in the worm (Wei et al., 2012). The activator (QF) contains a DNA-binding domain (aa1-183 [Riabinina et al., 2015]) that recognizes its cognate site QUAS in the QF responsive promoter, the latter also carrying *Δpes-10*, the minimal promoter from the worm gene *pes-10*. The QF activation domain (QFAD, aa650-816 [Riabinina et al., 2015]) can be inhibited by QS, and the effect is relievable by quinic acid (QA). The mechanism of QS inhibition is unknown (question mark), although a direct physical interaction is depicted. QF also carries a large “middle domain” (aa184-649) dispensable for activation (not shown) (Riabinina et al., 2015). (C and D) Tet/Q hybrid system, which comprises three components: a modified TRE bearing the minimal promoter *Δpes-10*, a modified rtTA called rtTA(Q) bearing QFAD, and a tet-regulated transcription silencer (tTS) consisting of TetR fused to the PIE-1 repressor domain. tTS and rtTA(Q) are co-expressed but omitted for clarity from Figures 1C and 1D, respectively.

Genetic tools for controlling transcription of engineered transgenes have revolutionized biomedical research and biotechnology. Among such tools, one of the most powerful is the Tetracycline (Tet) regulatory system, which allows efficient, graded, reversible, and spatiotemporal transcription regulation by Tet (Berens and Hillen, 2003, Schonig et al., 2010) (Figure 1A). The strength of the Tet system rests partly on the fact that its two prokaryotic components, the Tet repressor (TetR) and the tet operator (*tetO*), interact with exquisite specificity and prove ideally suited for functioning in eukaryotes. Furthermore, the two eukaryotic components of the Tet system, the VP16 activation domain and the CMV minimal promoter (Figure 1A), are also highly effective. Finally, tetracyclines and their analog Doxycycline (Dox) can readily permeate tissues and are well tolerated, and their pharmacology has been thoroughly analyzed because of their medical importance. The Tet system was originally developed (Gossen and Bujard, 1992) and subsequently used extensively for transgene regulation in mammalian cells and mice, with over 500 transgenic rodent lines already made by 2013 (Gossen and Bujard, 2002, Schonig et al., 2010, Schönig et al., 2013). It is also applicable to diverse lower organisms including *Drosophila* (Stebbins et al., 2001, McGuire et al., 2004, Ford et al., 2007), zebrafish (Knopf et al., 2010, Gu et al., 2013, Ma et al., 2017), *Xenopus* (Ridgway et al., 2000), chicken (Sato et al., 2002), and plant (Weinmann et al., 1994, Dutt et al., 2014). However, applications to the nematode *C. elegans*, a crucial model organism, are conspicuously absent.

Several alternative methods have been developed for controlling transcription in the worm. The simplest is to use tissue-specific promoters to effect spatial regulation, but temporal regulation is not feasible and neither is the expression level tunable. The same limitations apply to the second strategy, which uses a VP16-derived activation domain fused to GAL4 DNA-binding domain to control transgene expression (Wang et al., 2017). In the third strategy, heat shock is used to induce a transgene, but only transiently, as prolonged heating is lethal and even transient heating can potentially complicate the analysis. Furthermore, this method cannot achieve spatial regulation unless the experiment is done in the mutant defective in heat shock response everywhere except the cells of interest, but such a defect might have unintended consequences (Bacaj and Shaham, 2007). In the fourth strategy, a transcription termination cassette is placed upstream of the target gene; the cassette is removable and hence the target gene inducible upon the expression of site-specific recombinase such as Flp, which enables spatial regulation if the target transgene and/or recombinase are expressed from tissue-specific promoters. Furthermore, temporal control can be superimposed on the spatial regulation by expressing the recombinase from a heat-shocked promoter (Davis et al., 2008, Voutev and Hubbard, 2008), but transgene induction in this scenario is irreversible and non-tunable and its pattern confounded by the developmental history of the promoters that direct recombinase expression. The final strategy for transgene regulation in the worm is based on the Q system (Figure 1B), an elegant repressible binary expression system derived from the fungus *Neurospora crassa* (Riabinina and Potter, 2016). Like the Tet system, the Q system comprises the transactivator (called QF) and the responder (effector) that bears the transactivator-binding sites (called QUAS), but the system is unique in that QF is suppressible by its repressor QS, which can be relieved by quinic acid (QA), a non-toxic small molecule. Thus, QA can “induce” (actually de-repress) target genes in cells expressing both QF and QS, enabling spatial and temporal regulation (Wei et al., 2012). The Q system has also been applied to human cells, *Drosophila* (Potter et al., 2010), zebrafish (Subedi et al., 2014), and mosquito (Riabinina et al., 2016). However, compared with the Tet system, the Q system is used much less widely and its properties have been characterized much less extensively.

Here we describe a set of tools based on the Tet and Q systems that collectively enable highly efficient and versatile transgene regulation in the worm. At the heart of these innovations is a Tet/Q hybrid system that combines the strengths of the two systems (Figures 1C and 1D, see further).

## Results

### Adaption of the Tet System for the Worm

Our preliminary data indicate that the canonical Tet system is largely inactive in the worm. As shown in Figure 1A, in addition to the bacteria-derived rTetR protein and its DNA-binding sites (TetO), the Tet system contains two components derived from human viruses: the VP16 activation domain and the CMV minimal promoter. As both have evolved to interact with the human transcription machinery, they might function poorly in the worm. We first replaced the CMV minimal promoter in the Tet system with *Δpes-10*, the minimal promoter from the worm gene *pes-10* shown to be active in the worm (Wei et al., 2012). To evaluate the Tet system in diverse tissues, the modified responder (effector) was co-injected into the worm with a driver expressing rTetR-VP16 (rtTA) under the control of the broadly active *rpl-28* promoter *(Prpl-28)*.

**Figure 2:**
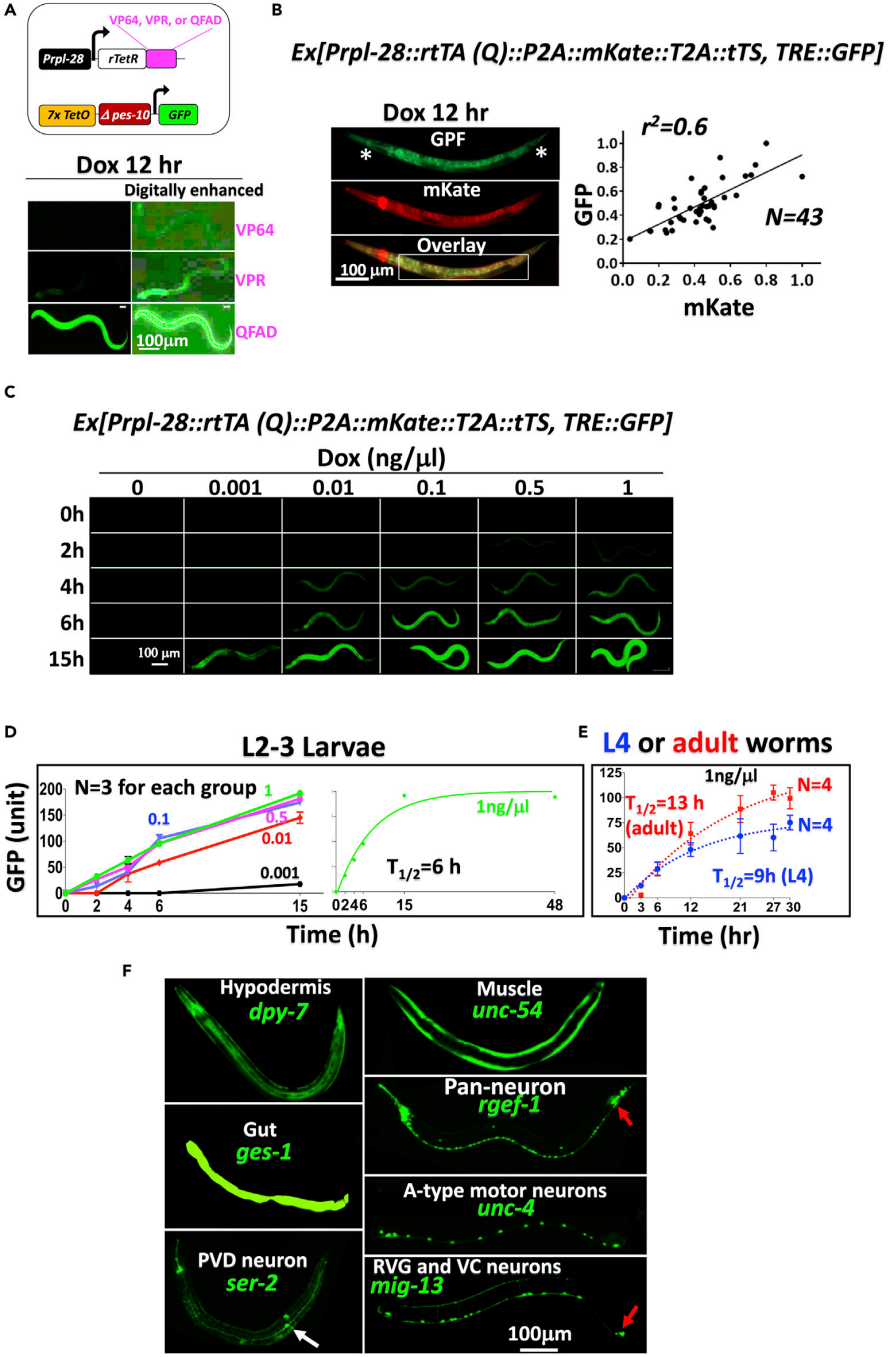
Development of the Tet/Q System (A) Comparison of activation domains. Transgenic larvae carrying Ex-arrays comprising the driver and responder plasmids depicted at the top were exposed to Dox and imaged under stereomicroscope (see Transparent Methods). GFP expression mediated by VP64 or VPR became visible only after digital overexposure (bottom right). (B) Robust GFP induction achieved using the Tet/Q system. (Left) Worms were imaged using upright microscope. The bright red fluorescence at the anterior is from the co-injection marker DsRed. The square at the bottom demarcates the area where fluorescence was quantified. Asterisks denote GFP expression without concomitant detectable mCherry signals at the first pharyngeal bulb and tail tip. These regions also tended to ectopically express GFP when rtTA(Q) was expressed from certain tissue-specific promoters (Figure 2F). However, worms carrying only the TRE-GFP transgene never express GFP at these regions (or anywhere else, for that matter). Thus, TRE seems hypersensitized at the pharyngeal bulb and tail tip, enabling GFP induction by rtTA even when its levels were below the detection limit. (Right) GFP vs. mKate expression on the demarcated area are plotted relative to the maximal signals (set as 1). Each dot represents a worm. Of note, in some worms, the mKate signal was enhanced following Dox stimulation, presumably reflecting unintended super-activation of *Prpl-28* by rtTA(Q) bound to nearby TRE in the Ex-arrays. (C–E) Kinetics and dose dependency of GFP induction. (C) L2-L3 larvae were transferred to plates containing increasing concentrations of Dox and imaged under stereomicroscope at various times thereafter. Three worms per Dox concentration were analyzed, with highly consistent results. (D) GFP intensity in the worm shown in Figure 2C was quantified and plotted as a function of time (left), where the numbers inside the plot denote Dox concentrations (ng/μL). The last time point for imaging was 15 hr after the initiation of Dox exposure, except that the worms exposed to the highest concentration of Dox (1 ng/μL) were additionally imaged at 48 hr. The data from the latter group of worms are employed to calculate T_1/2_ of GFP induction through non-linear curve fit (right). (E) Similar to D, but the experiments were done independently, using older worms (L4 larvae and Day 2 adults) and 1 ng/μL Dox. The dotted lines are fitted curves. Error bars in D and E, SEM. (F) Same as Figure 2B, except that the ubiquitous *Prpl-28* was replaced with various tissue-specific promoters and the worms were imaged under confocal microscope. The white and red arrows denote PVD neuron and ectopic expression in gut-like tissues in the tail region, respectively.

We found that the modified Tet system remained inefficient even when we replaced the VP16 activation domain in rtTA with VP64, the tetramerized minimal VP16 activation domain (Beerli et al., 1998) or VPR, the super-strong, tripartite activation domain comprising VP16 and two other mammalian activator domains (Chavez et al., 2015) (Figure 2A). In sharp contrast, robust GFP induction was seen when the VP16 activation domain was replaced with QFAD, the activation domain from the fungal transcription factor QF (Riabinina et al., 2015); the resulting rTetR-QFAD fusion protein will be termed rtTA(Q) hereafter (Figures 1C and 2A). Unfortunately, GFP expression was leaky. In this experiment, the driver and responder were co-injected and consequently co-exist on the same extrachromosomal arrays (Ex-arrays), which might cause the *Prpl-28* in the driver to inadvertently activate the responder via physical proximity. Alternatively, or additionally, the leakiness might reflect residual Dox-independent binding of rtTA(Q) to the Tetracycline Response Element (TRE), with the effect amplified by the highly potent QFAD and *pes-10* minimal promoter. In any case, the leakiness may be minimized (but induction is not compromised) using tet-regulated Transcription Silencer (tTS) comprising a repressor domain fused to TetR, which binds *tetO* only in the absence of Dox (Zhu et al., 2001). After testing several repressor domains, including the potent mammalian KRAB repressor domain, we found the most effective one to be that from PIE-1, a worm protein that blocks transcription by inhibiting Pol II carboxyl-terminal domain phosphorylation (Ghosh and Seydoux, 2008) (Figures 1D and S1A). This optimized binary system, which contains elements from both the Tet and the Q systems, is termed “Tet/Q hybrid system” (Figures 1C and 1D).

We next characterized the hybrid system in detail. Following 12 hr of Dox exposure (1 ng/μL), GFP was effectively induced throughout the body, although GFP intensities were rather punctate instead of uniform across various regions (Figure 2B, left, *GFP*). As rtTA expression level can dictate TRE activity (Katigbak et al., 2018, McJunkin et al., 2011), the variation in GFP expression might be caused by variable rtTA expression and thus reflected the property of *Prpl-28* rather than an inherent limitation of the hybrid system. This seems the case, because the GFP signals in general coincided with the mKate signals marking rtTA expression (Figure 2B, left, *Overlay*), with exceptions sometimes observed at the first pharyngeal bulb and tail tip (asterisks, Figure 2B). Averaged GFP intensities on individual worms are also well correlated with their respective mKate intensities (Figure 2B, scatterplot at the right).

We then examined the dose dependence of GFP induction kinetics and level in L2-3 larvae (Figure 2C). At the highest Dox concentrations (0.5–1 ng/μL), GFP became visible after a mere 2-hr treatment, whereas the kinetics was slower at lower doses. Besides, GFP fluorescence increased as a function of Dox concentration between 0.001 and 0.01 ng/μL, beyond which it plateaued. As expected, GFP was undetectable in the absence of Dox. The kinetics and extent of induction were reproducible (Figure 2D, left), with the T_1/2_ of GFP induction by 1 ng/μL Dox being 6 hr in the L2/3 larvae (Figure 2D, right). GFP induction became slower in older worms, with the T_1/2_ decreased to 9 and 13 hr for L4 larvae and Day 2 adults, respectively (Figure 2E).

Finally, to create reagents useful to the worm researchers, we constructed a set of driver plasmids using 7 popular tissue-specific promoters (McGhee and Krause, 1997) that are active in the hypodermis (*Pdpy7* [Gilleard et al., 1997]), gut (*Pges-1*) (Aamodt et al., 1991), muscle (*Punc-54*) (MacLeod et al., 1981), pan-neurons (*Prgef-1*) (Chen et al., 2011, p. 1), A-type motor neurons (*Punc-4*) (Miller and Niemeyer, 1995), PVD neurons (*ser-2*^*prom3*^) (Tsalik et al., 2003), and neurons in retrovesicular ganglion and the ventral cord (*Pmig-13*) (Sym et al., 1999), respectively. We confirmed that GFP expression patterns were in general agreement with the known promoter specificities. The GFP expression patterns among different worms in the same transgenic lines were comparable, although often not identical in terms of the exact sets of cells expressing GFP and/or the intensities of GFP signal within a particular cell (Figure S1B; see also Figure S3B). This variability resulted at least in part from the variability in rtTA(Q) expression driven by the tissue-specific promoters (Figure S1C), thus reflecting the properties of these promoters just as in the case of the variability in the GFP expression pattern controlled by *Prpl-28*-driven rtTA(Q) (Figure 2B). Of note, for *Prgef-1* and *Pmig-13*, ectopic induction was frequently observed within some gut-like structure in the tail region (arrows in Figure 2F), even though these regions at best weakly expressed rtTA(Q) (Figure S1C). Finally, leaky expression was undetectable except for ectopic weak signals in the tail region in a small fraction of the *Pmig-13* transgenic worms, suggesting that in the tail region, *Pmig-13* could overwhelm tTS to activate the adjacent TRE located on the same Ex-array (data not shown).

We conclude that the Tet/Q hybrid system enables fast, robust, dose-dependent, and tissue-specific transgene induction in the worm, with negligible leaky expression.

### *Inducible* Intersectional Techniques: Restricting Spatial Expression while Allowing Temporal Regulation

**Figure 3:**
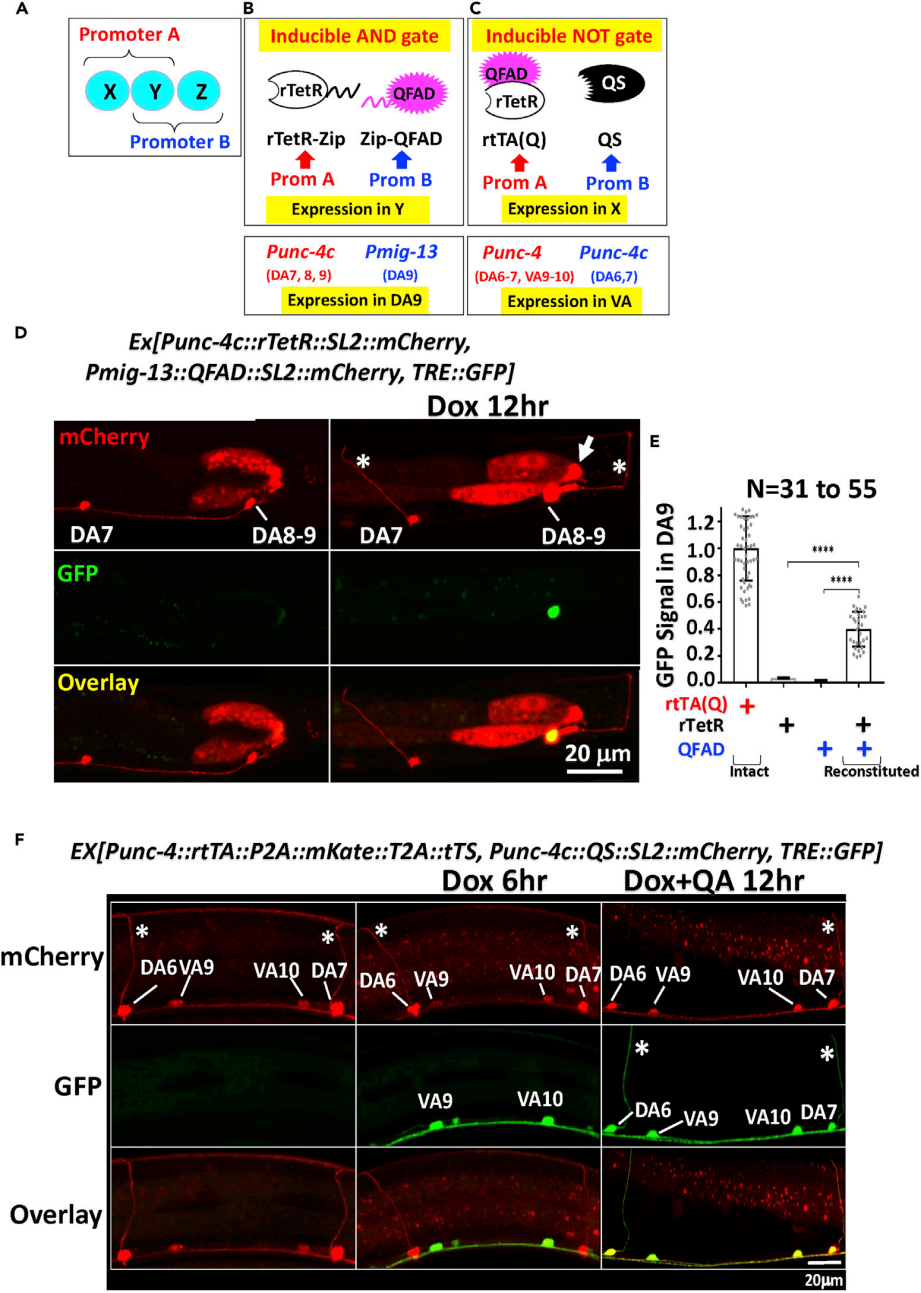
Inducible Intersectional Regulation (A–C) Experimental design. Hypothetical Promoters A and B with overlapping specificities (A) are used to direct the expression of individual components of the logic gates (B-C, top). The actual promoters used in this study and their respective relevant target cell types are listed at the bottom in Figures 3B and 3C. Note that, with this system, the expression is not only cell type specific but also Dox inducible. *Punc-4c*, a truncated *unc-4* promoter. (D and E) “AND” gate. (D) L4 larvae were treated as indicated before imaging GFP and mCherry expression in the tail region. Asterisks indicate the axonal commissures extending from the DA neurons to the dorsal nerve cord, which is diagnostic of DA neurons, as VA neurons lack such commissures. Arrows indicate ectopic expression. DA8 and DA9 neurons are overlapping and hard to distinguish (see, e.g., Figure 3E in reference Wei et al., 2012). (E) The activity of the reconstituted protein (as measured in the GFP intensity in the DA9 neuron) relative to that of the individual domains and the intact rtTA(Q) expressed from *Pmig-13*. Error bars, S.D. ****p < 0.0001, one-tailed t test. (F) “NOT” gate. L4 larvae were treated with drugs as indicated before imaging GFP and mCherry/mKate expression in the tail region. The asterisks indicate axonal commissures of the DA neurons. The DA neurons emitted brighter red fluorescence than VA neurons as they expressed both mKate (from *Punc-4*) and mCherry (from *Punc-4c*), whereas the VA neurons only expressed mKate (from *Punc-4*).

Promoters exclusively active in a particular cell population are often unavailable, as most promoters are expressed in multiple cell types. This is especially true for the nervous system because of its complexity and heterogeneity, which has constituted a bottleneck in deciphering neural circuits and behavior (Luo et al., 2008). An important strategy to gain genetic access to subsets of cells is to use intersectional methods, where two distinct promoters with overlapping expression patterns act combinatorially to restrict transgene expression via creation of logic gates (Zhang et al., 2004, Venken et al., 2011, Luo, 2015). Specifically, if promoter A is active in cell types X and Y and promoter B in Y and Z, then one may create an “AND” logic gate (if A and B, then C) for expression only in Y, or a “NOT” logic gate (If A but not B, then C) for expression only in X or Z (Figure 3A).

Our strategies for intersectional regulation are outlined in Figures 3B and 3C, which are similar to the strategies used to create logic gates based on the GAL4 and Q systems (Luan et al., 2006, Wei et al., 2012) (Wang et al., 2018). Thus, to create the AND gate, we split rtTA(Q) into the DNA-binding and transcription-activation domains (rTetR and QFAD, respectively) and fuse them to complementary leucine zippers. The two halves are then expressed from Promoters A and B, respectively, which should activate target genes only in cell type Y, where the two halves are co-expressed to reconstitute rtTA(Q) via leucine-zipper-mediated heterodimerization (Figure 3B). To create the NOT gate, rtTA(Q) and QS are expressed from promoters A and B, respectively, which should restrict target expression to cell type X. Importantly, since rTetR is subject to control by Dox, temporal control can be readily superimposed over spatial regulation, which is not feasible with conventional intersectional methods. For example, the NOT gate based on the Q system, in which promoters A and B express QFAD and QS, respectively, can restrict expression to cell type X, but the expression in this cell type is constitutive rather than inducible (Wei et al., 2012). The same limitation applies to an AND gate created by a split GAL4 system( Wang et al., 2018). We dub ours *inducible* intersectional method to emphasize its inherent temporal control capability.

AND and NOT logic gates have been created using the Q system to restrict GFP expression to specific subsets of the A type motor neurons in the posterior region of the ventral nerve cord in the worm (Wei et al., 2012). To facilitate direct comparison with the previous study, we employed the same promoters to create the logic gates and analyzed the same set of neurons. We were able to recapitulate the spatially restricted expression patterns (Wei et al., 2012), and, furthermore, we demonstrate that the expression was inducible by Dox for both types of logic gates.

#### Inducible AND Gate

*Punc-4c* and *Pmig-13* were used to express the two halves of rtTA(Q) in overlapping sets of neurons (DA7-9 vs. DA9; Figure 3B, bottom). All these neurons were labeled by mCherry co-expressed with the fusion proteins. Dox induced GFP only in DA9 (58%, 31/53) without any leaky expression (0/94) or ectopic induction (0/53) (Figure 3D). Based on the intensity of the GFP signals, the reconstituted protein was 40% as active as the intact rtTA(Q), whereas the individual domains were essentially inactive (Figure 3E). These results are comparable with those of the split QFAD system (Wei et al., 2012). Interestingly, in the tail region, the previous study found *Pmig-13* active in VA12 in addition to DA9 (Wei et al., 2012), whereas we could detect its activity only in DA9 (Figure 3D; see also Figure 2F). However, our observation is in apparent agreement with another study (Figure 5G in reference Sym et al., 1999). Some subtle differences in the driver constructs might underlie the discrepancies.

#### Inducible NOT Gate

*Punc-4c* and *Punc-4* were used to express QS and rtTA(Q) in the overlapping sets of neurons (DA6-7 vs. DA6-7+ VA9-10). In worm expressing both proteins, a 6-hr Dox exposure sufficed to induce significant GFP in the VA neurons (Figure 3F, middle column) without ectopic expression in the DA neurons even after 12-hr Dox exposure (not shown); such a fast induction echoes that seen in the worms expressing rtTA from *Prpl-28* (Figure 2C). As expected, DA neurons expressed GFP following simultaneous exposure to Dox and QA, but consistent with previous observations (Wei et al., 2012), the induction kinetics was slower, with significant expression seen in the majority of DA neurons (96%, 65/68) only after 12 hr of treatment (Figure 3F, right column).

### A QS-Refractory QFAD Mutant Enables Independent Manipulation of Distinct Transgenes in the Same Cells

Sophisticated genetic analysis can entail independent manipulation of different cell populations in the organism or different genes in the same cells. The simultaneous use of the hybrid and Q systems would enable the former manipulation but not the latter. This is because rtTA(Q) and QF both use QFAD as the activation domain, and QS, intended for controlling QF, would inevitably inactivate rtTA(Q) at the same time, if rtTA (Q) is expressed in the same cells as QF. To address this problem, we searched for QS-resistant QFAD mutants. QF is structurally similar to GAL4 (Riabinina et al., 2015), and our bioinformatics analysis revealed a short stretch of conserved sequence between the activation domains of the two proteins (Figure S2A). In particular, QFAD (781–786) is homologous to GAL4 (864–869) known to be essential for GAL80 to bind and suppress GAL4 (Wu et al., 1996). Furthermore, QFAD (781–786) is predicted to be part of an alpha helix (Figure S2B), suggesting its involvement in interaction with other proteins such as QS. Extensive mutagenesis indicates that deleting QFAD (778–789) indeed made QFAD refractory to QS, but without compromising its activation potential (Figure S2C). This rtTA(Q) deletion mutant will be called rtTA(Q*) hereafter.

**Figure 4:**
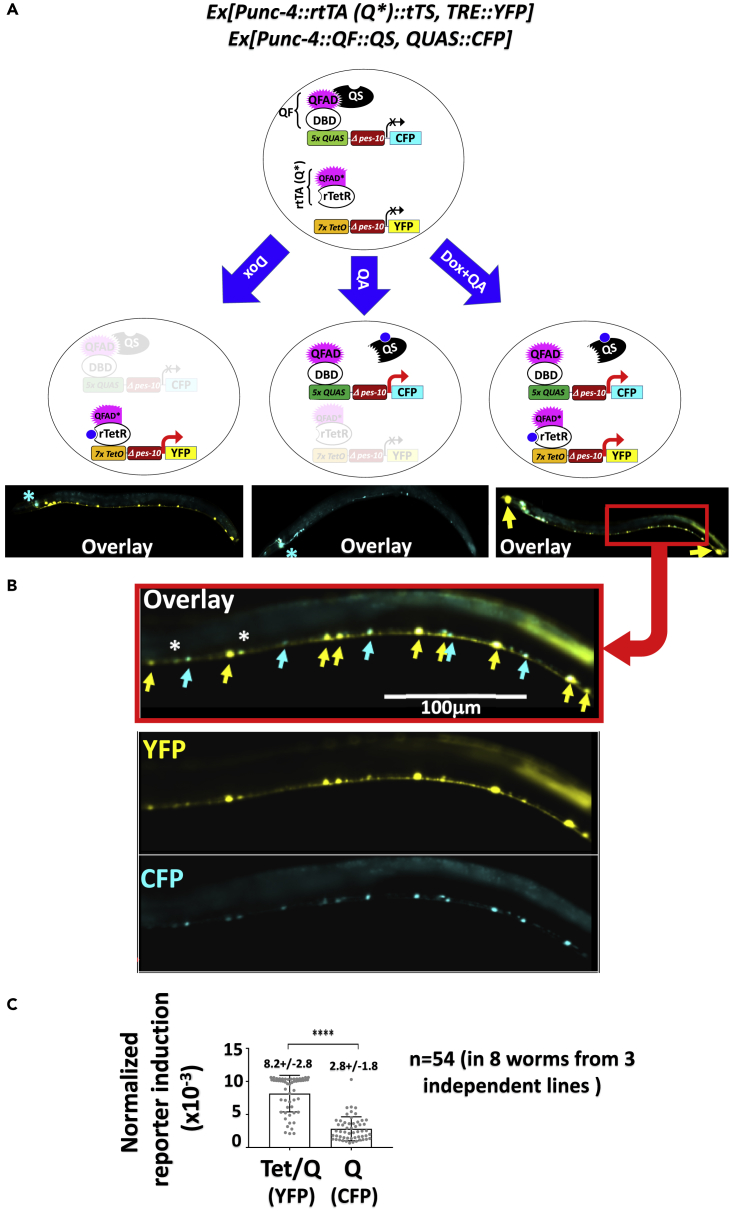
Independent Regulation of Distinct Transgenes in the Same Cells (A and B) Young adults doubly transgenic for the Tet/Q and Q systems were exposed to the indicated inducers for 24 hr before imaging (0.1″ and 1″ exposures for YFP and CFP, respectively). In Figure 4A, the asterisks in the left and middle images denote the signals perhaps derived partly from the co-injection marker GFP expressed from the *Odr-1* promoter, whereas the yellow arrows in the image at the right indicate ectopic YFP expression in the first pharyngeal bulb and tail tip observed in some worms (see also Figures 2B and 2F). In Figure 4B, where the portion within the red square in Figure 4A is displayed together with the images of the worm in the YFP and CFP channels, the asterisks indicate neurons with comparable YFP vs. CFP expression, whereas yellow and cyan arrows indicate neurons with predominant YFP and CFP fluorescence, respectively. (C) Tet/Q is 3-fold more active than Q. The YFP and CFP signals in the experiment described in Figure 4B, which reflect the inducibility of the Tet/Q and Q systems, respectively, are quantified, normalized by intrinsic differences in the brightness of the two fluorescence proteins, and plotted. A total of 54 neurons in 8 worms derived from 3 independently generated lines were analyzed. The images were captured and quantified using Zeiss ZEN 2.3 lite and the values scored when both fluorescence signals in the same neurons were detectable and within the lineage range (determined in Figure S3C). Compared with CFP, the YFP exposure time was 10× shorter but fluorescence 14× stronger (Figure S3D), and so the raw values of YFP signals were divided by 1.4 before plotting. Error bars, S.D. ****p < 0.0001, two-tailed t test.

To test the feasibility of independent regulation of distinct transgenes in the same cells, we first made two Ex-array transgenic lines carrying either the Q or the modified hybrid system, which used different reporters (CFP or YFP, respectively) in the responders but the same promoter (*Punc-4*, active in A type motor neurons) in the drivers. CFP or YFP was undetectable at the resting state (not shown) but clearly induced by QA and Dox, respectively, with the two reporters showing no spectral overlap (Figure S3A). We then mated the two lines to obtain double transgenic offspring and analyzed their responses to Dox/QA stimulation. There was no CFP or YFP expression in the absence of inducers (not shown), whereas Dox and QA indeed independently controlled their respective target genes in the neurons (Figures 4A and 4B). Thus, the two systems can function orthogonally, as long as rtTA(Q*) is used.

### Tet/Q Is More Robust than Q

In the above-described double transgenic worms co-expressing YFP and CFP, the YFP and CFP signals were somewhat variable in different A type motor neurons within the same worms and in the same neurons in different worms (Figures 4B and S3B), which presumably reflects (partly) the property of *Punc4* used to direct rtTA(Q*) and QF expression. Importantly, the YPF signal in general appeared much brighter and was visible in more neurons than the CFP signal, even when the exposure time for YFP imaging was set 10× shorter than for CFP (0.1″ vs. 1”). This holds true for three independent double transgenic lines examined, suggesting that the Tet/Q hybrid system, which used YFP as the reporter, can more effectively induce target genes than the Q system. However, YFP may be intrinsically more stable and/or brighter than CFP. To address this caveat, we made transgenic lines carrying an Ex-array comprising the *Punc-4*-rtTA(Q) driver and two responders that were identical except for the fluorescent proteins expressed (YFP vs. CFP). Following Dox stimulation, the worms were imaged as in Figure 4B and neuron fluorescence quantified. We scored those neurons whose YFP and CFP intensities were both within the lineage range (pre-determined in Figure S3C) and found the YFP signal 14× stronger (Figure S3D). Using this value for normalization, we inferred that the Dox can activate reporter expression to a level ∼3-fold higher than that achieved by QA-mediated de-repression, indicating that Tet/Q is more robust than the Q system (Figure 4B).

### Robust Control of Cre Expression Using the Tet/Q Hybrid System

Finally, we attempted to use the hybrid system to control Cre expression as an example of its applications. Cre was selected because of its importance in biotechnology. In addition, Cre leaky expression and ectopic/insufficient induction can be detected quite reliably using sensitive reporter/PCR assays that examine the consequences of Cre-mediated recombination. Cre thus offers a highly stringent test for the robustness of our system.

**Figure 5:**
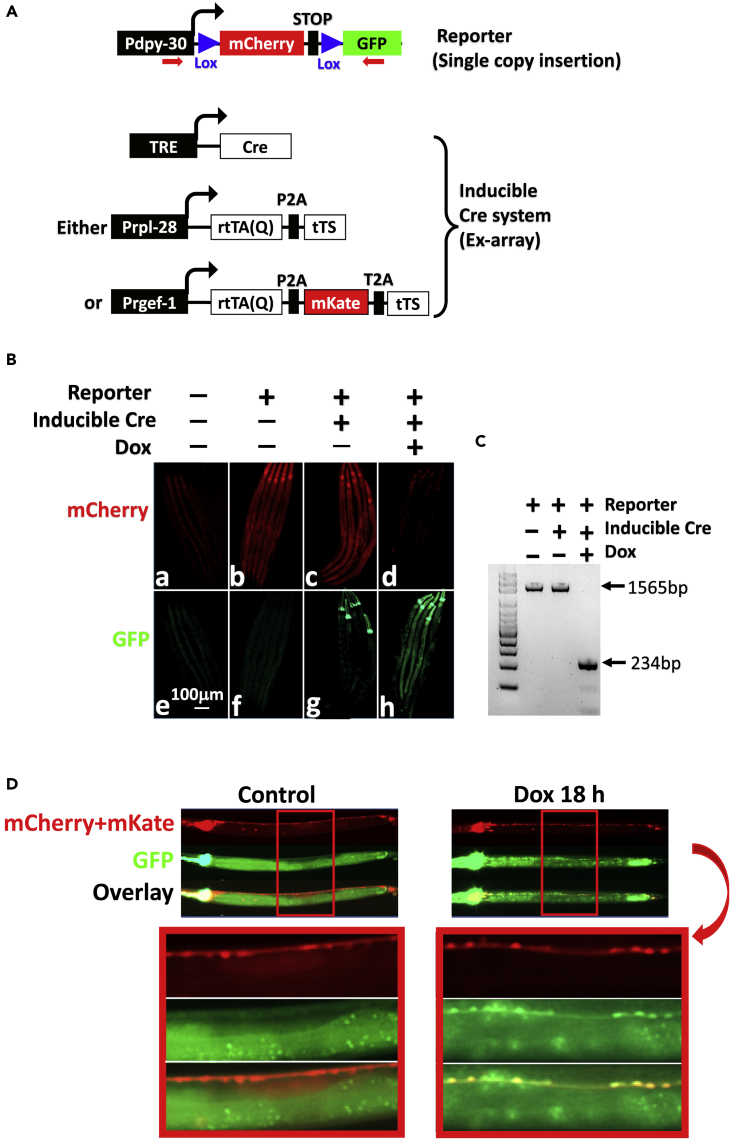
Robust Regulation of Cre Expression (A) Transgenes used in the study. The Cre reporter is randomly integrated while the Cre inducible systems carried on Ex-arrays. rtTA(Q) is expressed from either the ubiquitous *Prpl-28* or neuron-specific *Prgef-1,* which was used for experiments described in Figures 5B–5D, respectively. In the latter case, mKate is co-expressed with rtTA(Q) to label neurons. Red arrows indicate PCR primers for detecting Cre-mediated excision of the floxed cassette in the Cre reporter as described in Figure 5C. (B and C) Effects of global Cre induction controlled by rtTA(Q) widely expressed from *Prpl-28.* Adult worms were transferred to Dox plates, and 2 days later, young adult F1 worms were imaged (Figure 5B, last column, showing 5 worms) and genomic DNA was analyzed by PCR for Cre-mediated deletion (Figure 5C) using the primers depicted in Figure 5A. Control worms were similarly processed. mCherry and GFP were imaged with 4.2″ and 3.5″ exposures, respectively. The bright green signals at the anterior were from the co-injection marker GFP. At least 10 worms in each group were imaged, with consistent results. (D) Dox induced GFP specifically in the neurons when rtTA(Q) was expressed from *Prgef-1*. mKate was co-expressed with rtTA(Q) to label the neurons. The mCherry widely expressed from the single copy reporter template was much weaker than the mKate expressed from the multi-copy Ex-array and basically invisible under the imaging condition used, which explains why the red fluorescence was restricted to neurons.

The constructs for the experiment are depicted in Figure 5A. The Cre reporter carries a floxed mCherry-terminator cassette inserted between *Pdpy-30* (a constitutive, ubiquitous promoter) and *GFP*, so that mCherry is widely expressed until Cre excises the terminator cassette to switch the expression to GFP. The reporter was stably integrated into the genome as a single-copy gene using miniMos1 technology (Frokjaer-Jensen et al., 2014) rather than carried in an Ex-array, because Cre-mediated deletion is expected to dramatically shorten, and hence potentially eliminate, the Ex-array (Mello et al., 1991). rtTA(Q) was expressed from either the broadly active *Prpl-28* or neuron-specific *Prgef-1*, allowing the evaluation of Cre induction in diverse tissues and in a specific cell type, respectively. The drivers were introduced into the Cre-reporter line in conjunction with TRE-Cre responder (Figure 5A).

We first examined the effects of ubiquitous Cre induction (Figure 5B). Adult worms with various genotypes as indicated were allowed to lay eggs on plates lacking (first three column) or containing (last column) Dox. Two days after hatching, the F1 young adults were imaged. We found that the reporter line expressed mCherry but not GFP, as expected (Figure 5B, image *b, f*). The Ex-array did not alter reporter expression in the absence of Dox (image c, *g*) but eliminated mCherry while switching on GFP following 2 days of Dox exposure (image *d, h*). PCR analysis of the Cre reporter transgene confirmed that recombination was undetectable in the control worm but complete after Dox exposure (Figure 5C). In these experiments, GFP signals were already apparent within 24 hr of Dox exposure but the mCherry signal lingered on for one more day presumably reflecting the slow decay of preexisting mCherry molecules (not shown).

We next examined the effects of Cre induction in neurons in the worms expressing rtTA(Q) from *Prgef-1*. To unambiguously identify the neurons, we co-expressed mKate with rtTA(Q) (Figure 5A). GFP was undetectable in the neurons in the absence of Dox but clearly induced within 18 hr of Dox exposure in all the mKate-expressing cells, indicating that Cre-mediated deletion was already complete by this time (Figure 5D).

These data illustrate the utility of the Tet/Q system for tight and effective control of biologically important genes.

## Discussion

A prominent feature of the Tet/Q system is its robustness, with rapid, strong, dose-dependent induction and negligible leakiness. The system is far more active than the canonical Tet system in the worm. Indeed, we found that VP16-based activation domains function poorly in the worm, which may explain why the canonical Tet system has never been described in the worm. Interestingly, several studies have reported the use of VP16-based activation domains in the worm. First, “VP160,” which comprises 10 tandem copies of VP16 activation domains, is fused to dCAS9 to upregulate target gene expression, but even with the simultaneous use of six sgRNAs targeting the same gene, only a mild (∼3×) effect can be achieved (Long et al., 2015). In the second study, VP64 is fused to GAL4 DNA-binding domain to activate transcription from a responder bearing GAL4-binding sites (Wang et al., 2017). Here as many as 15 copies of the GAL4-binding sites are needed to support optimal activation, which might have compensated for the weakness in the VP64 activation potential. Since QFAD is far more active than VP64 and presumably also VP160 in the worm, its use might help perfect the two gene regulatory tools mentioned earlier. Of note, the GAL4 system is not inducible, which limits its applications.

Tet/Q is also more robust than the Q system. This observation is not surprising, as previous studies have demonstrated that QA is unable to fully de-repress QF. Specifically, in the worm, QF expressed in DA and VA neurons robustly activates a GFP reporter in all these neurons in 80% of the worms, which is efficiently suppressed by QS. However, QA exposure for 24 hr can de-repress GFP in all these neurons in only 40% of the worms (Figure 1o in Wei et al., 2012). Similarly, even with a 30-hr incubation, QA can only partially de-repress GFP in the body wall muscle in the worm expressing QF in this tissue, with the GFP signal much weaker than observed in the worm lacking QS (Figure S3 in Wei et al., 2012). In the *Drosophila* S2 cells, QA seems even less effective, rescuing only 10% of QF activity (Figure S1 in Potter et al., 2010). Given the inability of QA to fully inactivate QS, the QF activity rescued is expected to be lower than that induced by Dox, assuming that Dox can effectively activate rTetR in the worm as in other organisms. It would be interesting to engineer a QS mutant that is more responsive to QA to optimize this elegant binary system. It is also noteworthy that even the current version of the Q system can function effectively on single-copy reporter genes (Wei et al., 2012), suggesting the same for the Tet/Q system, which should broaden its applications.

Robust binary systems like the Tet/Q system have the potential for widespread use in the worm. First, they are fit for demanding applications such as tight regulation of Cre expression. Furthermore, libraries of drivers and responder lines can be separately created and then systematically combined by breeding, allowing one driver to control diverse responders and vice versa, which would greatly facilitate genetic analysis. Currently, worm researchers typically resort to tissue-specific promoters to directly control transgene expression, which requires the tedious processes of plasmid injection and worm characterization for each new construct. This situation may change following the adoption of the binary inducible systems, as exemplified in *Drosophila* where thousands of GAL4 driver lines and a wide selection of reporter lines are available, which has helped revolutionize the field (Southall et al., 2008).

The Tet/Q system is not only robust but also highly flexible. First, it is subject to dual control by two distinct inducers (Dox and quinic acid), which is unique among binary gene regulatory systems, as other systems are either not inducible (e.g., the GAL4-based system) or controlled by a single inducer (e.g., the Tet or Q system). Second, we have created the split Tet/Q system and the QS-resistant QFAD mutants, which adds to its flexibility. The Tet/Q system and its modifications, in conjunction with the Q system, enable sophisticated modes of transgene manipulation, such as inducible intersectional regulation and independent control of distinct transgenes in the same cells. These strategies will expand the range of applications of both the Tet/Q and the Q systems.

We have also briefly explored the reversibility of GFP induction in the experiments described in Figure 2C. Specifically, the worms were exposed to Dox (1ng/ul) for 15 hr, washed several times, and transferred to a fresh plate.

GFP signal persisted for at least 2 days without any sign of decay (data not shown), presumably because the washes were insufficient to deplete Dox from the worm. More extensive washes, combined with lower Dox concentration (e.g., 0.01ng/ul) and shorter exposure times (e.g., 6 hr) might help facilitate the reversal.

As mentioned in the *Introduction*, the Tet system has been reported to function in diverse lower organisms. However, such reports are sporadic, in contrast to the scenario in mammalian cells. Given that the Tet system was originally developed for mammalian cells, its performance in the lower organisms might be suboptimal, thus preventing their widespread use. This issue might be addressed by replacing the mammalian elements in the system (the VP16 activation domain and the CMV minimal promoter) with those highly active in the respective lower organisms, as illustrated in our study.

Finally, although this study focuses on transcription regulation, gene expression can also be controlled at the protein level, and auxin-inducible protein degradation (Zhang et al., 2015) has been achieved in the worm, which can complement transcription manipulation for sophisticated control of gene expression.

### Limitations of the Study

All of the transgenic lines except the Cre reporter line described in Figure 5A were generated using microinjection of plasmids into the gonads, which produces complex Ex-arrays that are known to be unstable and susceptible to silencing. To facilitate the use of our system in the worm, it will be helpful to generate driver and responder lines carrying single-copy transgenes stably integrated into the genome.

## Methods

All methods can be found in the accompanying Transparent Methods supplemental file.

## Data and Software Availability

All data generated or analyzed in this study are included in this published article (and its Supplemental Information files). The strains and plasmids will be deposited at CGC (https://cgc.umn.edu/) and Addgene (https://www.addgene.org/), respectively.

## Supplemental Information

Document S1. Transparent Methods and Figures S1–S3

## References

[bib1] Aamodt E.J., Chung M.A., McGhee J.D. (1991). Spatial control of gut-specific gene expression during *Caenorhabditis elegans* development. Science.

[bib2] Bacaj T., Shaham S. (2007). Temporal control of cell-specific transgene expression in *Caenorhabditis elegans*. Genetics.

[bib3] Beerli R.R., Segal D.J., Dreier B., Barbas C.F. (1998). Toward controlling gene expression at will: specific regulation of the erbB-2/HER-2 promoter by using polydactyl zinc finger proteins constructed from modular building blocks. Proc. Natl. Acad. Sci. U S A.

[bib4] Berens C., Hillen W. (2003). Gene regulation by tetracyclines. Constraints of resistance regulation in bacteria shape TetR for application in eukaryotes. Eur. J. Biochem..

[bib5] Chavez A., Scheiman J., Vora S., Pruitt B.W., Tuttle M., Iyer P.R., Lin S., Kiani S., Guzman C.D., Wiegand D.J. (2015). Highly efficient Cas9-mediated transcriptional programming. Nat. Methods.

[bib6] Chen L., Fu Y., Ren M., Xiao B., Rubin C.S. (2011). A RasGRP, C. elegans RGEF-1b, couples external stimuli to behavior by activating LET-60 (Ras) in sensory neurons. Neuron.

[bib7] Davis M.W., Morton J.J., Carroll D., Jorgensen E.M. (2008). Gene activation using FLP recombinase in *C. elegans*. PLoS Genet..

[bib8] Dutt M., Dhekney S.A., Soriano L., Kandel R., Grosser J.W. (2014). Temporal and spatial control of gene expression in horticultural crops. Hortic. Res..

[bib9] Ford D., Hoe N., Landis G.N., Tozer K., Luu A., Bhole D., Badrinath A., Tower J. (2007). Alteration of Drosophila life span using conditional, tissue-specific expression of transgenes triggered by doxycyline or RU486/Mifepristone. Exp. Gerontol..

[bib10] Frokjaer-Jensen C., Davis M.W., Sarov M., Taylor J., Flibotte S., LaBella M., Pozniakovsky A., Moerman D.G., Jorgensen E.M. (2014). Random and targeted transgene insertion in *C. elegans* using a modified Mosl transposon. Nat. Methods.

[bib11] Ghosh D., Seydoux G. (2008). Inhibition of transcription by the *Caenorhabditis elegans* germline protein PIE-1: genetic evidence for distinct mechanisms targeting initiation and elongation. Genetics.

[bib12] Gilleard J.S., Barry J.D., Johnstone I.L. (1997). cis regulatory requirements for hypodermal cell-specific expression of the *Caenorhabditis elegans* cuticle collagen gene dpy-7. Mol. Cell. Biol..

[bib13] Gossen M., Bujard H. (1992). Tight control of gene expression in mammalian cells by tetracycline-responsive promoters. Proc. Natl. Acad. Sci. U S A.

[bib14] Gossen M., Bujard H. (2002). Studying gene function in eukaryotes by conditional gene inactivation. Annu. Rev. Genet..

[bib15] Gu Q., Yang X., He X., Li Q., Cui Z. (2013). Generation and characterization of a transgenic zebrafish expressing the reverse tetracycline transactivator. J. Genet. Genomics.

[bib16] Katigbak A., Robert F., Paquet M., Pelletier J. (2018). Inducible genome editing with conditional CRISPR/Cas9 mice. G3 (Bethesda).

[bib18] Knopf F., Schnabel K., Haase C., Pfeifer K., Anastassiadis K., Weidinger G. (2010). Dually inducible TetON systems for tissue-specific conditional gene expression in zebrafish. Proc. Natl. Acad. Sci. U S A.

[bib19] Long L., Guo H., Yao D., Xiong K., Li Y., Liu P., Zhu Z., Liu D. (2015). Regulation of transcriptionally active genes via the catalytically inactive Cas9 in C. elegans and D. rerio. Cell Res..

[bib20] Luan H., Peabody N.C., Vinson C.R., White B.H. (2006). Refined spatial manipulation of neuronal function by combinatorial restriction of transgene expression. Neuron.

[bib21] Luo L. (2015). Principles of Neurobiology.

[bib22] Luo L., Callaway E.M., Svoboda K. (2008). Genetic dissection of neural circuits. Neuron.

[bib23] Ma Z., Zhu P., Pang M., Guo L., Chang N., Zheng J., Zhu X., Gao C., Huang H., Cui Z. (2017). A novel inducible mutagenesis screen enables to isolate and clone both embryonic and adult zebrafish mutants. Sci. Rep..

[bib24] MacLeod A.R., Karn J., Brenner S. (1981). Molecular analysis of the unc-54 myosin heavy-chain gene of *Caenorhabditis elegans*. Nature.

[bib55] McGhee J.D., Krause M.W., Riddle D., Blumenthal T., Meyer B., Priess J. (1997). Analysis of *C. elegans* Promoters. C. *elegans* II, Vol. 33.

[bib25] McGuire S.E., Roman G., Davis R.L. (2004). Gene expression systems in Drosophila: a synthesis of time and space. Trends Genet..

[bib26] McJunkin K., Mazurek A., Premsrirut P.K., Zuber J., Dow L.E., Simon J., Stillman B., Lowe S.W. (2011). Reversible suppression of an essential gene in adult mice using transgenic RNA interference. Proc. Natl. Acad. Sci. U S A.

[bib27] Mello C.C., Kramer J.M., Stinchcomb D., Ambros V. (1991). Efficient gene transfer in *C. elegans*: extrachromosomal maintenance and integration of transforming sequences. EMBO J..

[bib29] Miller D.M., Niemeyer C.J. (1995). Expression of the unc-4 homeoprotein in *Caenorhabditis elegans* motor neurons specifies presynaptic input. Development.

[bib31] Potter C.J., Tasic B., Russler E.V., Liang L., Luo L. (2010). The Q system: a repressible binary system for transgene expression, lineage tracing and mosaic analysis. Cell.

[bib32] Riabinina O., Luginbuhl D., Marr E., Liu S., Wu M.N., Luo L., Potter C.J. (2015). Improved and expanded Q-system reagents for genetic manipulations. Nat. Methods.

[bib33] Riabinina O., Task D., Marr E., Lin C.C., Alford R., O'Brochta D.A., Potter C.J. (2016). Organization of olfactory centres in the malaria mosquito *Anopheles gambiae*. Nat. Commun..

[bib34] Riabinina O., Potter C.J. (2016). The Q-System: a versatile expression system for drosophila. Methods Mol. Biol..

[bib36] Ridgway P., Quivy J.P., Almouzni G. (2000). Tetracycline-regulated gene expression switch in *Xenopus laevis*. Exp. Cell Res..

[bib37] Sato N., Matsuda K., Sakuma C., Foster D.N., Oppenheim R.W., Yaginuma H. (2002). Regulated gene expression in the chicken embryo by using replication-competent retroviral vectors. J. Virol..

[bib38] Schonig K., Bujard H., Gossen M. (2010). The power of reversibility regulating gene activities via tetracycline-controlled transcription. Methods Enzymol..

[bib39] Schönig K., Freundlieb S., Gossen M. (2013). Tet-Transgenic Rodents: a comprehensive, up-to-date database. Transgenic Res..

[bib40] Southall T.D., Elliott D.A., Brand A.H. (2008). The GAL4 system: a versatile toolkit for gene expression in Drosophila. CSH Protoc..

[bib41] Stebbins M.J., Urlinger S., Byrne G., Bello B., Hillen W., Yin J.C. (2001). Tetracycline-inducible systems for Drosophila. Proc. Natl. Acad. Sci. U S A.

[bib42] Subedi A., Macurak M., Gee S.T., Monge E., Goll M.G., Potter C.J., Parsons M.J., Halpern M.E. (2014). Adoption of the Q transcriptional regulatory system for zebrafish transgenesis. Methods.

[bib43] Sym M., Robinson N., Kenyon C. (1999). MIG-13 positions migrating cells along the anteroposterior body axis of *C. elegans*. Cell.

[bib44] Tsalik E.L., Niacaris T., Wenick A.S., Pau K., Avery L., Hobert O. (2003). LIM homeobox gene-dependent expression of biogenic amine receptors in restricted regions of the *C. elegans* nervous system. Dev. Biol..

[bib45] Venken K.J.T., Simpson J.H., Bellen H.J. (2011). Genetic manipulation of genes and cells in the nervous system of the fruit fly. Neuron.

[bib46] Voutev R., Hubbard E.J.A. (2008). A “FLP-Out” system for controlled gene expression in *Caenorhabditis elegans*. Genetics.

[bib47] Wang H., Liu J., Gharib S., Chai C.M., Schwarz E.M., Pokala N., Sternberg P.W. (2017). cGAL, a temperature-robust GAL4-UAS system for *Caenorhabditis elegans*. Nat. Methods.

[bib48] Wang H., Liu J., Yuet K.P., Hill A.J., Sternberg P.W. (2018). Split cGAL, an intersectional strategy using a split intein for refined spatiotemporal transgene control in *Caenorhabditis elegans*. Proc. Natl. Acad. Sci. U S A.

[bib49] Wei X., Potter C.J., Luo L., Shen K. (2012). Controlling gene expression with the Q repressible binary expression system in *Caenorhabditis elegans*. Nat. Methods.

[bib50] Weinmann P., Gossen M., Hillen W., Bujard H., Gatz C. (1994). A chimeric transactivator allows tetracycline-responsive gene expression in whole plants. Plant J..

[bib51] Wu Y., Reece R.J., Ptashne M. (1996). Quantitation of putative activator-target affinities predicts transcriptional activating potentials. EMBO J..

[bib52] Zhang L., Ward J.D., Cheng Z., Dernburg A.F. (2015). The auxin-inducible degradation (AID) system enables versatile conditional protein depletion in *C. elegans*. Development.

[bib53] Zhang S., Ma C., Chalfie M. (2004). Combinatorial marking of cells and organelles with reconstituted fluorescent proteins. Cell.

[bib54] Zhu Z., Ma B., Homer R.J., Zheng T., Elias J.A. (2001). Use of the tetracycline-controlled transcriptional silencer (tTS) to eliminate transgene leak in inducible overexpression transgenic mice. J. Biol. Chem..

