# Supplementary material for: A Tet/Q Hybrid System for Robust and Versatile Control of Transgene Expression in C. elegans
Source: iScience. 2018 Dec 27;11:224–37. doi: 10.1016/j.isci.2018.12.023 (PMC6327101; doi:10.1016/j.isci.2018.12.023)
Supplement: Document S1. Transparent Methods and Figures S1–S3 [file mmc1.pdf]

ISCI, Volume *11*

Supplemental Information

A Tet/Q Hybrid System for Robust and Versatile Control of Transgene Expression in *C. elegans*

Shaoshuai Mao, Yingchuan Qi, Huanhu Zhu, Xinxin Huang, Yan Zou, and Tian Chi

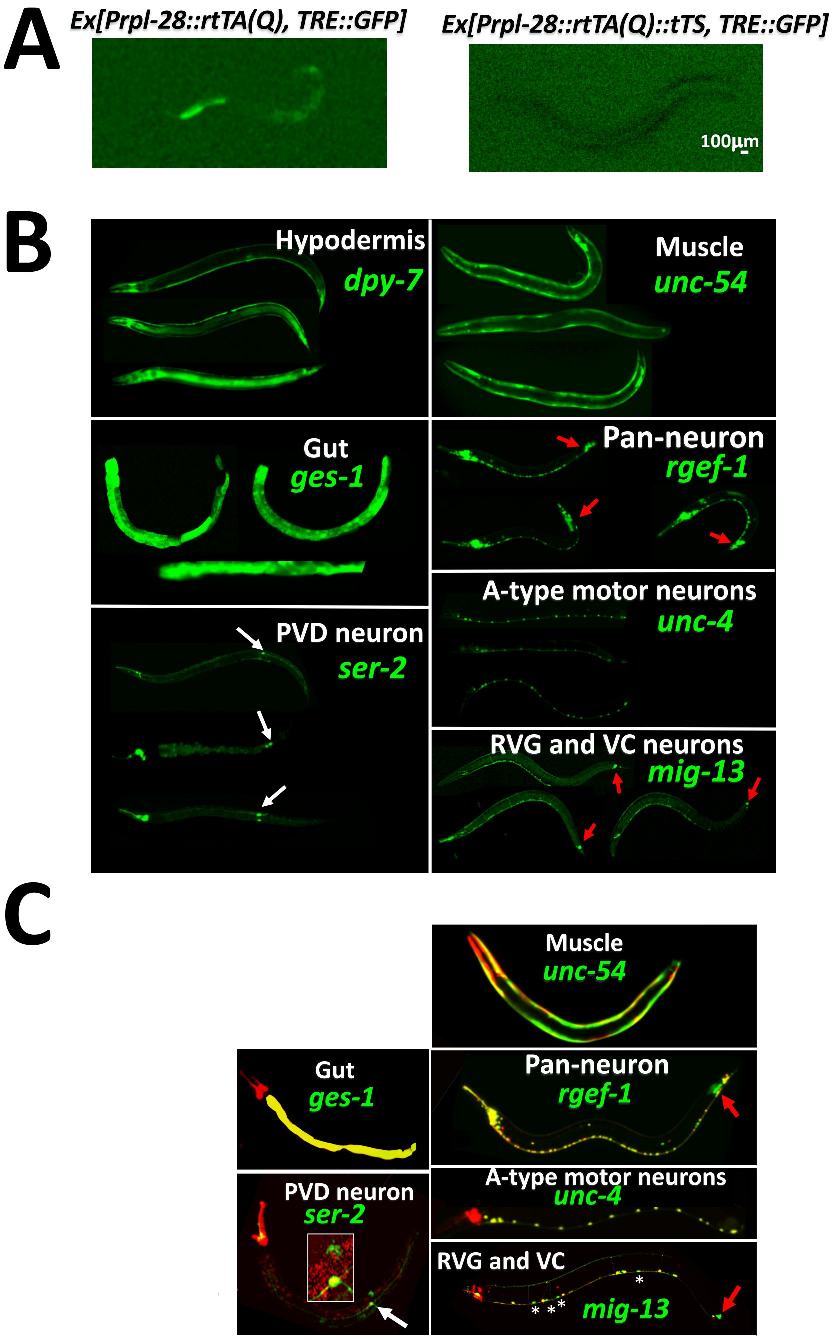

Fig. S1. Development of the Tet/Q system, related to Fig. 2

(A) Elimination of leaky expression using tTS. L4 larvae carrying indicated transgenes that lack (left) or express tTS (right) were compared. The images were digitally enhanced to reveal leaky expression.

(B) Similarity of GFP expression patterns in different worms of the same strain. Three worms for each line are displayed. The while and red arrows denote PVD neuron and ectopic expression in gut-like tissues in the tail region, respectively.

(C) Tight correlation of mKate v.s GFP expression in the majority of the transgenic strains. The images are the same as in Fig. 2F except that mKate signals, a proxy for rtTA(Q) expression, are overlaid with GFP signals. mKate expression driven by the *ges-1, ser-2 , rgef-1* and *unc-4* promoters was well correlated with GFP signals. Curiously, in the worm expressing mKate from the *unc-54* and *mig-13* promoters , multiple regions showed discordance of the mKate and GFP signals, which has two types of manifestations. First, in both lines, several regions/cells expressed GFP but little mKate (e.g., the cells marked by asterisks in mig-13 transgenic line), and the exact regions/cells involved were divergent among different worms of the same strain. However, the GFP expression here were apparently not ectopic, suggesting rtTA(Q) was correctly expressed despite the lack of its proxy mKate, an idea testable by direct detection of rtTA(Q) protein. The second manifestation of the mKate-GFP disconnect is that in the worm expressing rtTA(Q) from the *unc-54* promoter, mKate was expressed in several regions without concomitant GFP signal, and except for the head, the regions involved tend to differ among different individuals. The mechanism is unclear, but might involve some repressors of rtTA(Q) and/or TRE that stochastically silenced GFP. The white arrow at the bottom left indicates the PVD neuron, with the GFP signal above representing ectopic expression and the inset highlighting the mKate-GFP correlation (the weak mKate signal was digitally enhanced in the inset for better visualization). The red arrows in the right panels denote ectopic expression at the tail tip. Of note, the data on hypodermis is not shown because instead of mKate, BFP, which proved undetectable, was used for monitoring rtTA(Q) expression.

A

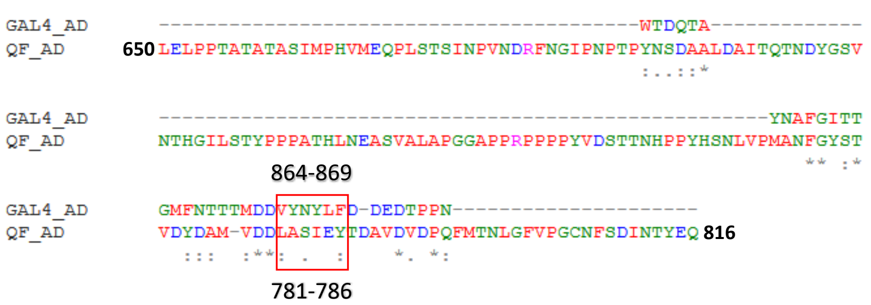

B

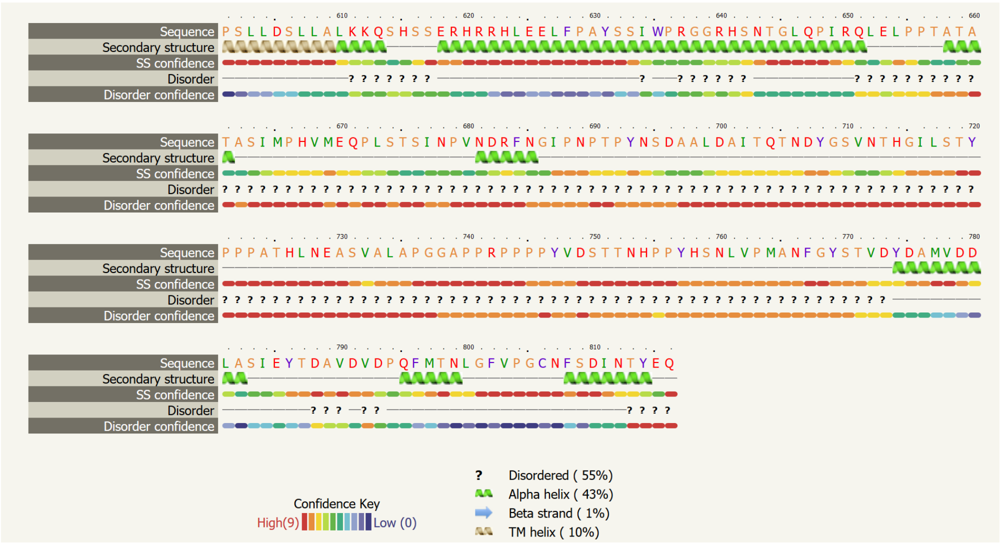

C

QFAD protein	QS repression	Activation
650-816	+	+++
650-768	-	+
650-777	-	+
650-768+790-816	-	++
650-777+790-816	-	+++
650-768+778-816	+	++
L781E	+	+++

Fig. S2. Identification of QS-resistant QFAD mutants, related to Fig. 4.
(A) Sequence alignment between QFAD (namely, aa 650-816 in QF) (Riabinina *et al.*, 2015) and the GAL4 activation domain, performed using Clustal Omega(https://www.ebi.ac.uk/Tools/msa/clustalo/). Boxed are the GAL4 residues important for GAL80 suppression and the corresponding residues in QFAD. The three icons underneath the amino acid sequences (:, ., w) denote strongly conservative changes, weakly conservative changes and identical residues, respectively.
(B) QFAD secondary structure prediction by Phyre2(Kelley and Sternberg, 2009)
(C) Summary of mutagenesis experiments. The point mutation L781E was tested because L868E in GAL4 is known to make GAL4 refractory to GAL80; L868 is located in the conserved region between GAL4 and QFAD (Fig. 4A)(Wu, Reece and Ptashne, 1996).

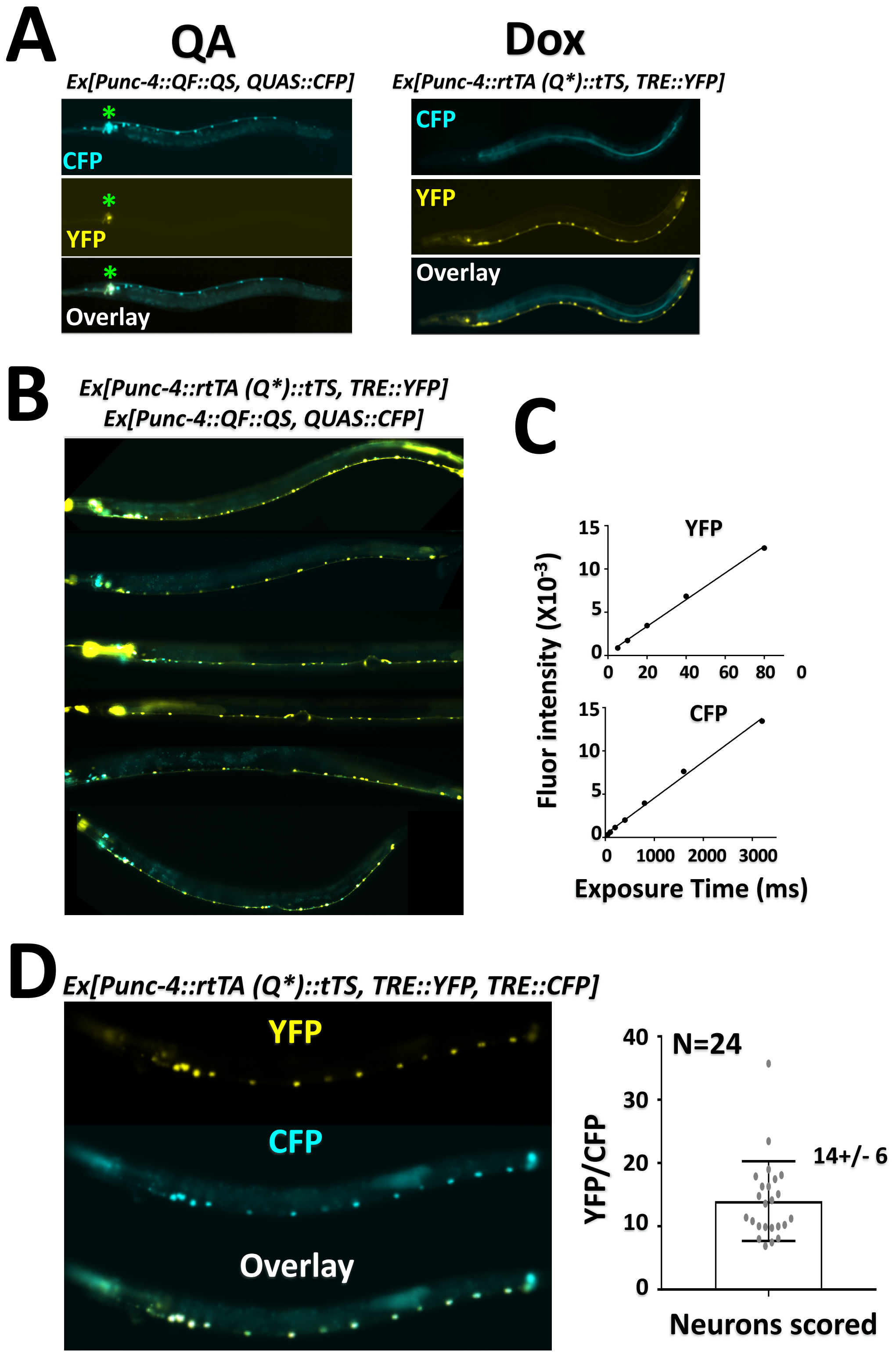

Fig. S3. The Tet/Q system is 3x more potent than the Q system, related to Fig. 4.

(A) No spectral overlap between CFP and YFP. Single array worms transgenic for the Q and Tet/Q system were induced with QA and Dox, respectively, for 24 hr before imaging. Asterisks denote the signals perhaps derived partly from the co-injection marker GFP expressed from the *Odr-1* promoter (GFP signals can leak into the CFP and YFP channels).

(B) YFP and CFP signals, driven by *Punc4,* are somewhat variable among individual neurons and worms. Shown are 6 worms derived from 3 independent lines. The images are overlays of YFP and CFP fluorescence.

(C) Determination of the lineage range of YFP and CFP imaging. On a double transgenic worm co-expressing the two proteins described in Fig. 4, a single neuron was sequentially imaged with various exposure times as indicated. The result shows that fluorescence intensities below 15,000 units are within the lineage range of the detection system. Images were captured and quantified using Zeiss ZEN 2.3 lite.

(D) Determination of intrinsic differences between YFP and CFP. The worm line carried an equal quantity of *TRE-YFP* and *TRE-CFP* responders, which were activated simultaneously in A type-motor neurons by rtTA(Q*) expressed from *Punc-4*. Images were captured and quantified as described in Fig. S3C, with the exposure times set at 0.1' and 1' for YFP and CFP, respectively (left). The neurons where both YFP and CFP signals were clearly detectable and within the lineage range were scored. To calculate the relative YFP/CFP intensity within a neuron, the raw YFP value was multiplied by 10 before division by the CFP value in the same cell. A total of 24 neurons from 3 independently derived worm lines were analyzed (right).

TRANSPARENT METHODS

Expression constructs

Plasmids were made using pPD95_77 as the backbone (a gift from Andrew Fire, Addgene plasmid # 1495), with the relevant protein coding sequences codon-optimized for worm expression. Detailed cloning strategies are described below, and important plasmids will be deposited at Addgene.

TC358 TRE-Δpes-10-GFP: 7xTetO was amplified from TC243 with primer 5'- AATAAGCTTGCATGCCCGTCTTCACTCGAGTTT and 3'- CACGCCTACCTCGACA, Δpes-10 minimal promoter was amplified from N2 genomic DNA with primer 5'- GTCGAGGTAGGCGTGATCGATTTTTTGCAAATTACGAG and 3'- ACTCATTTTTTCTACCGGCTGAAAGTTAAAAATTACAGT. 7x TetO and Δpes-10[reference] were inserted into AgeI-PstI fragment from 95.77_GFP_WITH_K from Huan Hu using In-Fusion HD cloning kit(Clontech).

TC549 Prpl-28::rtetR-VPR::P2A::mKate::T2A::tetR-pie1: VPR was codon-optimized by IDT and synthesized by GeneWiz, P2A::mKate::T2A::tetR-pie1 and Prpl-28::rtetR were amplified from TC374 with 5'- CCATGGGGCAGTGGGGCTA and 3'- ACCGGAACCAGATCCAGATCCAG. Two fragments were fused using In-Fusion HD cloning kit(Clontech).

TC357 Prpl-28::rtetR-QFAD::P2A::mKate: rtetR-QFAD::P2A was codon-optimized and synthesized by GenScript, mKate was amplified from pPD285 mKate2_C1_SE with 5'- ATGGTCTCCGAGCTCATT and 3'- GCGCTCAGTTGGAATTACGGTGTCCGAGCTTG. rtetR-QFAD::P2A and mKate were inserted into EcoRI-BamHI fragment from TC350 rpl-28p_in_pPD95 using In-Fusion HD cloning kit(Clontech).

TC374 Prpl-28::rtetR-QFAD::P2A::mKate::T2A::tetR-pie1: rtetR-QFAD::P2A::mKate was amplified from TC357 with 5'- GACGCTCTCGTGGATCAAA and 3'- ACGGTGTCCGAGCTTG. T2A::tetR-pie1 was codon-optimized and synthesized. rtetR-QFAD::P2A::mKate and T2A::tetR-pie1 were inserted into EcoRI-BamHI fragment from TC350 rpl-28p_in_pPD95 using In-Fusion HD cloning kit(Clontech).

TC393 Pser-2::rtetR-QFAD::P2A::mKate::T2A::tetR-pie1: Ser-2 promoter was released with BamHI and NotI digestion Pser-2 promoter-mCherry. rtetR-QFAD::P2A::mKate::T2A::tetR-pie1 was amplified from TC374 with 5'- GCGCCTCTAGAGGATCGCTAGCACGCTCTCGTGGATCAAA and 3'- CCAGTCAGTGCGGCCGCGGTAATACGGTTATCCACA. ser-2 promoter was subcloned into TC374 to replace the rpl-28 promoter using In-Fusion HD cloning kit(Clontech).

TC414 Prpl-28::rtetR-QFAD::P2A::eBFP2::T2A::tetR-pie1: eBFP2 was codon optimized and synthesized and amplified with 5'- GAGAACCCTGGACCTGT and 3'- TCCCTCTCCCGATCCT. T2A::tetR-pie1::Prpl-28::rtetR-QFAD::P2A was amplified from TC374 with 5'- GGATCGGGAGAGGGACGAGGAAGT and 3'- AGGTCCAGGGTTCTCCTCCACGTCT. The two fragments were infused using In-Fusion HD cloning kit(Clontech).

TC419 Pges-1::rtetR-QFAD::P2A::mKate::T2A::tetR-pie1: Ges-1 promoter was amplified from 95.77-kp-ges-1-prom-raga-1-DA with 5'- CGTATTACCGCGGCCGCAACATCACGGACCCAA and 3'- CGAGAGCGTGCTAGCTCTAGAGTCGACTGAATTCAA, then subcloned into the NotI and NheI sites of TC393, replacing the ser-2 promoter.

TC426 Pdpy-7::rtetR-QFAD::P2A::eBFP2::T2A::tetR-pie1: dpy-7 promoter was released with NotI and BamHI from pSM Pdpy-7_lin-14a_Cflag. rtetR-QFAD::P2A::eBFP2::T2A::tetR-pie1 was amplified from TC414 with 5'- GCGCCTCTAGAGGATCGCTAGCACGCTCTCGTGGATCAAA and 3'- CCAGTCAGTGCGGCCGCGGTAATACGGTTATCCACA. The two fragments were fused using In-Fusion HD cloning kit(Clontech).

TC474 Prgef-1::rtetR-QFAD::P2A::mKate::T2A::tetR-pie1 rgef-1 promoter was amplified from worm gDNA with 5'- ggccgcactgactggTGCAGGCAATACTAATAGAGG and 3'- atcctctagaggcgcTTTTGGATCCCGTCGTC. The vector was amplified from TC374 with 5'- gcgcctctagaggatcGCTAGCACGCTCTCGTGGATCAAA and 3'- ccagtcagtgcggccGCGGTAATACGGTTATCCACA. Two fragments were fused using In-Fusion HD cloning kit(Clontech).

TC476 Punc-54::rtetR-QFAD::P2A::mKate::T2A::tetR-pie1: unc-54 promoter was amplified from pSM-Punc-54_mcherry with 5'- GGCCGCACTGACTGGGCCGGCCAAGCTTGTCTT and 3'- ATCCTCTAGAGGCGCGCTAGCCAAGGGTCCTC. rtetR-QFAD::P2A::mKate::T2A::tetR-pie1 was amplified from TC374 with 5'- GCGCCTCTAGAGGATCGCTAGCACGCTCTCGTGGATCAAA and 3'- CCAGTCAGTGCGGCCGCGGTAATACGGTTATCCACA. Two fragments were fused using In-Fusion HD cloning kit(Clontech).

TC512 Pmig-13::Zip(-)::QFAD::NLS::SL2::mCherry: mig-13 promoter was amplified from N2 genomic DNA with 5'- CTTGCATGCGCGGCCGGTACCCTGACACTAAGTTTC and 3'- CAATTGTTCAGAAGCCATTACCTGAAATTCTGAATTAAATGAT. Zip(-) was codon-optimized and synthesized by GenScript and then amplified with 5'- GCTTCTGAACAATT and 3'- GGTACCTGATCCA. QFAD was amplified from TC357 with 5'- TCTGGATCAGGTACCCTTGAGCTTCCGCCGA and 3'- TTAGACCTTACGCTTCTTCTTTGGCCCACTGCCCCATG. SL2 was amplified from N2 genomic DNA with 5'- AAGCGTAAGGTCTAAGCTGTCTCATCCTACTTTC and 3'- CCATTTTTTCTACCGGATGCGTTGAAGCAGTT. Four fragments were inserted into NotI and KpnI site of pSM-Punc-54_mcherry to replace the unc-54 promoter using ClonExpress MultiS One Step Cloning Kit(Vazyme).

TC513 Punc-4c::SV40 NLS::rtetR::Zip(+)::SL2::mCherry: unc-4c promoter was amplified from N2 genomic DNA with 5'- CTTGCATGCGCGGCCGCTGACTGGTGATCCATCTCG and 3'- GACCTTACGCTTCTTCTTTGGCATTTTCACTTTTTGGAA. rtetR was amplified from TC357 with 5'- AAGAAGCGTAAGGTCATGTCACGACTGGATAAAT and 3'- AGATCCTGATCCGGATCCTCCTCC, Zip(+)::SL2 was codon-optimized and synthesized. The three fragments were inserted into NotI and KpnI site of pSM-Punc-54_mcherry (to replace unc-54 promoter) using ClonExpress MultiS One Step Cloning Kit(Vazyme).

TC520 Punc-4c::QS::SL2::mCherry: ordered from addgene(XW09)(Wei *et al.*, 2012)

TC544 Prpl-28::rtetR-VP64::SV40 NLS::P2A::mKate::T2A::tetR-pie1: VP64::SV40 NLS was codon-optimized and synthesized. P2A::mKate::T2A::tetR-pie1 and Prpl-28::rtetR were amplified from TC374 with 5'- CCATGGGGCAGTGGGGCTA and 3'- ACCGGAACCAGATCCAGATCCAG. The two fragments were fused using In-Fusion HD cloning kit(Clontech).

TC550 Punc-4::rtetR-QFAD::P2A::mKate::T2A::tetR-pie1: unc-4 promoter was amplified from N2 genomic DNA with 5'- AACCGTATTACCGCGGCCGCCAACAATATAGGATGCTCAG and 3'- TTTATCCAGTCGTGACATTTTCACTTTTTGGAAGA, rtetR-QFAD::P2A::mKate::T2A::tetR-pie1 was amplified from TC374 with 5'- ATGTCACGACTGGATAAATCGAA and 3'- GCGGCCGCGGTAATACGGTTATCCACA. The two fragments were fused using In-Fusion HD cloning kit(Clontech).

TC560 Punc-4::rtetR-QFAD(650-768)::P2A::mKate::T2A::tetR-pie1 fragment deleted aa778-789 was amplified from TC550 with 5'- CCATGGGGCAGTGGGGCTA and 3'- CCCACTGCCCCATGGATATCCGAAATTGG then self-infused using In-Fusion HD cloning kit(Clontech).

TC561 Punc-4::rtetR-QFAD(650-768)::P2A::mKate::T2A::tetR-pie1 fragment deleted aa778-789 was amplified from TC550 with 5'- CCATGGGGCAGTGGGGCTA and 3'- CCCACTGCCCCATGGCACCATCGCATCATAGTCTACAGTC, then fused using In-Fusion HD cloning kit(Clontech).

TC562 Punc-4::rtetR-QFAD(650-777+790-816)::P2A::mKate::T2A::tetR-pie1 fragment deleted aa778-789 was amplified from TC550 with 5'- GTTGACGTTGATCCGCAGTTCA and 3'- CGGATCAACGTCAACCACCATCGCATCATAGTCTACAGTC then self-infused using In-Fusion HD cloning kit(Clontech).

TC574 Punc-4::rtetR-QFAD(650-768)::P2A::mKate::T2A::tetR-pie1 fragment deleted aa778-789 was amplified from TC550 with 5'- GTTGACGTTGATCCGCAGTTCA and 3'- TGCGGATCAACGTCAACATATCCGAAATTGGCCATGGG then fused using In-Fusion HD cloning kit (Clontech).

TC608 Punc-4::rtetR-QFAD(650-768+778-816)::P2A::mKate::T2A::tetR-pie1 fragment deleted aa778-789 was amplified from TC550 with 5'- GCCAATTTCGGATATGATGATCTGGCATCGATCGAGT and 3'- ATATCCGAAATTGGCCATGGGGAC then fused using In-Fusion HD cloning kit(Clontech).

TC646 Pdpy30::loxP::mcherry::let-858 terminator::loxP::GFP. This construct was codon optimized and synthesized by Genscript, also named as “pQA1321”.

TC672 TRE::CFP CFP was amplified from TC812 with 5’- gacaccATGGTTTCTAAGGGAGAAGAACTTTT and 3’- AGACTTTTTTCTTGGCGGCAC. The vector was amplified from TC358 with 5’- ccaagaaaaaagtctCCGACACCCGCCAAC and 3’- AGAAACCATGGTGTCTCTCCAATCTCC. Two fragments were fused using In-Fusion HD cloning kit(Clontech).

TC690 Punc4::QF2::QS::mKate QS was amplified from TC520 to make TC674(unc4::rtTA::QF2::QS::mKate::Tts, QF2 was synthesized by Genscript). 2 fragments (unc-4 promoter and QF2::P2A::QS::P2A::mKate) were amplified from TC674 with 5’- AAGCTCGGACACCGTTGAGCGCCGGTCGCTA 3’- CATTTTCACTTTTTGGAAGAAGAAGATCCTC and 5’- CAAAAAGTGAAAATGCCGCCTAAACGCAAGAC 3’- ACGGTGTCCGAGCTTGGATGGGA. Two fragments were fused using In-Fusion HD cloning kit(Clontech).

TC708 Pmig-13::rtetR-QFAD::P2A::mKate::T2A::tetR-pie1: mig-13 promoter was amplified from TC512 with 5’- TGCTGATAAATCTGGAGCCGGTGA and 3’- TTTATCCAGTCGTGACATTACCTGAAATTCTGAATTAAATGAT, rtetR-QFAD::P2A::mKate::T2A::tetR-pie1 was amplified from TC374 with 5’- ATGTCACGACTGGATAAATCGAA and 3’- TCACCGGCTCCAGATTTATCAGCAA. The two fragments were fused using In-Fusion HD cloning kit(Clontech).

TC709 Punc-4::rtetR-QFAD(L781E)::P2A::mKate::T2A::tetR-pie1 fragment deleted aa778-789 was amplified from TC550 with 5’- TGTAGACTATGATGCGATGGTGGATGATGAGGCATCGATCGAGTA and 3’- TGTAGACTATGATGCGATGGTGGATGATGAGGCATCGATCGAGTA then fuse using In-Fusion HD cloning kit(Clontech).

TC811 TRE::YFP YFP was from PBS77-ChR2(H134R)::YFP(from Yan Zou), which was digest and inserted into BsaI and NcoI sites of TC358 using NEB enzymes.

TC812 QUAS::CFP 5xQUAS and CFP was codon optimized and synthesized by Genscript. Fragment was amplified with 5’- CGCTAACAACTTGGAAATGAAAT and 3’- TTATTTATACAATTCATCCATTCCA. Vector was amplified from TC358 with 5’- GAATTGTATAAATAAGCATTCGTAGAATTCCAACTGAG and 3’- GGCATGCAAGCTTATTTCATTTCC. Two fragments were fused using In-Fusion HD cloning kit(Clontech).

TC872 TRE::nCre nCre was codon optimized and synthesized by genscript and amplified with 5’- gtaatttttaactttcagaaggacccaaaggtatgtttcgaatg and 3’- AGACTTTTTTCTTGGCGGCAC, the vector was amplified from TC358 with 5’- ccaagaaaaaagtctCTCTGACACATGCAGCTCC and 3’- ctgaaagttaaaaattac. Two fragments were fused using In-Fusion HD cloning kit(Clontech).

Strains and transformation

All transgenic lines carrying Ex-arrays were generated on the N2 background using standard protocols (Mello and Fire, 1995). At least 3 transgenic lines for each transformation were obtained. The line bearing the single copy Cre reporter insertion (Fig. 5) was generated using the mini-mos1 strategy (Frokjaer-Jensen *et al.*, 2014). Strain information is described below.

**Plasmid mixture injected**	**Expts**
ms18 (VP64) [TC544(5ng/ul), TC358(5ng/ul), Podr-1::dsRED(50ng/ul), NEB 1kb DNA ladder(40ng/ul)].	Fig.2A
ms22 (VPR) [TC549(5ng/ul), TC358(5ng/ul), Podr-1::dsRED(50ng/ul), NEB 1kb DNA ladder(40ng/ul)]	Fig.2A
ms15 (QFAD) [TC357(5ng/ul), TC358(5ng/ul), Podr-1::dsRED(50ng/ul), NEB 1kb DNA ladder(40ng/ul)]	Fig.2A, Fig.S1A
ms11 (rtTA(Q) + tTS) [TC374(5ng/ul), TC358(5ng/ul), Podr-1::dsRED(50ng/ul), NEB 1kb DNA ladder(40ng/ul)]	Fig.2B-C, Fig.S1A

ms08 (dpy-7) [TC426(5ng/ul), TC358(5ng/ul), Podr-1::dsRED(50ng/ul), NEB 1kb DNA ladder(40ng/ul)]	Fig.2F, Fig.S1B
ms09 (rgef-1) [TC474(1ng/ul), TC358(5ng/ul), Podr-1::dsRED(50ng/ul), NEB 1kb DNA ladder(40ng/ul)]	Fig.2F, Fig.S1B
ms10 (unc-54) [TC476(1ng/ul), TC358(5ng/ul), Podr-1::dsRED(50ng/ul), NEB 1kb DNA ladder(40ng/ul)]	Fig.2F, Fig.S1B
ms12 (ser-2) [TC393(5ng/ul), TC358(5ng/ul), Podr-1::dsRED(50ng/ul), NEB 1kb DNA ladder(40ng/ul)]	Fig.2F, Fig.S1B
ms16 (ges-1) [TC419(5ng/ul), TC358(5ng/ul), Podr-1::dsRED(50ng/ul), NEB 1kb DNA ladder(40ng/ul)]	Fig.2F, Fig.S1B
ms73 (mig-13) [TC708(10ng/ul), TC358(5ng/ul), Podr-1::dsRED(50ng/ul), NEB 1kb DNA ladder(35ng/ul)]	Fig.2F, Fig.S1B, Fig.3E
ms77 (unc-4) [TC550(5ng/ul), TC358(5ng/ul), Podr-1::dsRED(50ng/ul), NEB 1kb DNA ladder(40ng/ul)]	Fig.2F, Fig.S1B
ms03 (and gate) [TC512(10ng/ul), TC513(30ng/ul),TC358(5ng/ul), Podr-1::dsRED(50ng/ul), NEB 1kb DNA ladder(5ng/ul)]	Fig.3D, 3E
ms74 ("and gate" control) [TC512(10ng/ul), TC358(5ng/ul), Podr-1::dsRED(50ng/ul), NEB 1kb DNA ladder(35ng/ul)]	Fig.3E
ms75 ("and gate" control) [TC513(30ng/ul),TC358(5ng/ul), Podr-1::dsRED(50ng/ul), NEB 1kb DNA ladder(15ng/ul)]	Fig.3E
ms100 ("not gate") [TC550(10ng/ul), TC520(10ng/ul),TC358(5ng/ul), Podr-1::dsRED(50ng/ul), NEB 1kb DNA ladder(25ng/ul)]	Fig.3F
ms119 (orthogonal) [TC562(5ng/ul), TC811(10ng/ul), Podr-1::dsRed(50ng/ul), NEB 1kb DNA ladder(35ng/ul)]	Fig.4, S3A, S3B
ms120 (orthogonal) [TC690(10ng/ul), TC812(10ng/ul), Podr-1::GFP(50ng/ul), NEB 1kb DNA ladder(30ng/ul)]	Fig.4, S3A, S3B
ms132 (cre) ex[TC414(5ng/ul), TC872(5ng/ul), Podr-1::GFP(50ng/ul), NEB 1kb DNA ladder(40ng/ul)]; si[pQA 1321]	Fig.5B, 5C
ms128 (cre) ex[TC474(5ng/ul), TC872(5ng/ul), Podr-1::GFP(50ng/ul), NEB 1kb DNA ladder(40ng/ul)]; si[pQA 1321]	Fig.5D
ms23 (mapping) ex[TC550(5ng/ul), TC520(5ng/ul), TC358(5ng/ul), Podr-1::dsRED(50ng/ul), NEB 1kb DNA ladder(35ng/ul)]	Fig.S2C
ms24 (mapping) ex[TC560(5ng/ul), TC520(5ng/ul), TC358(5ng/ul), Podr-1::dsRED(50ng/ul), NEB 1kb DNA ladder(35ng/ul)]	Fig.S2C
ms25 (mapping) ex[TC561(5ng/ul), TC520(5ng/ul), TC358(5ng/ul), Podr-1::dsRED(50ng/ul), NEB 1kb DNA ladder(35ng/ul)]	Fig.S2C
ms26 (mapping) ex[TC562(5ng/ul), TC520(5ng/ul), TC358(5ng/ul), Podr-1::dsRED(50ng/ul), NEB 1kb DNA ladder(35ng/ul)]	Fig.S2C
ms27 (mapping) ex[TC574(5ng/ul), TC520(5ng/ul), TC358(5ng/ul), Podr-1::dsRED(50ng/ul), NEB 1kb DNA ladder(35ng/ul)]	Fig.S2C
ms28 (mapping) ex[TC608(5ng/ul), TC520(5ng/ul), TC358(5ng/ul), Podr-1::dsRED(50ng/ul), NEB 1kb DNA ladder(35ng/ul)]	Fig.S2C
ms29 (mapping) ex[TC709(5ng/ul), TC520(5ng/ul), TC358(5ng/ul), Podr-1::dsRED(50ng/ul), NEB 1kb DNA ladder(35ng/ul)]	Fig.S2C
ms131 (YFP/CFP) ex[TC574(5ng/ul), TC672(5ng/ul), TC811(5ng/ul), NEB 1kb DNA ladder(85ng/ul)]	Fig.S3D

Drug treatment

30mg Dox (Doxycycline hyclate, ≥98%, Sigma-Aldrich D9891) was dissolved in 60 ul DMSO and then diluted to 1ml with water to get 30mg/ml stock solution. Before use, the stock was diluted to 1 ng/ul with M9 buffer, and 400ul was added onto each nematode growth medium (NGM) plate (60× 15 mm^2) seeded with *Escherichia coli* OP50, reaching the final concentration of 0.1ng/ul (except in Fig. 2E as indicated). This concentration is much lower than used in mammalian cell culture and no toxicity on the worm was observed. QA (at the final concentration of 7.5ug/ul) was used on NGM plate exactly as described (Wei *et al.*, 2012).

Fluorescence imaging and image quantification

The worms imaged in this study were L4 larvae or young adults unless specified otherwise. The worms in Fig. 2A, Fig. 2C and Fig. S1 were imaged with Nikon smz25 stereomicroscope (equipped with a SHR Plan Apo 1.6x objective and a DS-Ri1-U3 camera) using NIS-Elements software. All other images were captured with a Zeiss Axio Imager Z2 upright microscope (equipped with EC Plan-Neofluar 10x/0.30, Plan-Apochromat 20x/0.8, Plan-Apochromat 63x/1.4 Oil objectives, Axiocam506 camera, and Apotome.2) , except that the images in Fig. 2F and Fig. 3 were captured with Nikon A1R confocal microscope (equipped with S Plan Fluor ELWD 20x/0.45, Plan Fluor 40x/1.3 Oil, Apo TIRF 60x/1.49 Oil). Confocal images were rendered by maximum intensity projection method using Image J (US National Institutes of Health). Where applicable, images captured with the upright microscope were quantified using Zen 2.3 lite (Carl Zeiss), whereas confocal images with NIS-Elements software. Except for stereomicroscopy, the worms were immobilized using 10mM levamisole before imaging on 2% agarose pad.
